# Supplementary material for: End Sequence Analysis Toolkit (ESAT) expands the extractable information from single-cell RNA-seq data
Source: Genome Res. 2016 Oct;26(10):1397–410. doi: 10.1101/gr.207902.116 (PMC5052061; doi:10.1101/gr.207902.116)
Supplement: Supplemental Material [file supp_gr.207902.116_Supp_Materials.tar.gz › ESAT-esat_v0.1/doc/html/classbroad_1_1core_1_1datastructures_1_1_pair_3_01_t1_01_4-members.html]

ESAT: Member List


ESAT


- Main Page
- Related Pages
- Packages
- Classes
- Files

- Class List
- Class Index
- Class Hierarchy
- Class Members

All Classes Namespaces Files Functions Variables Enumerator Pages

- broad
- core
- datastructures
- Pair< T1 >

broad.core.datastructures.Pair< T1 > Member List

This is the complete list of members for broad.core.datastructures.Pair< T1 >, including all inherited members.

|  |  |  |
| --- | --- | --- |
| equals(Pair< T1 > other) | broad.core.datastructures.Pair< T1 > |  |
| equals(Object other) | broad.core.datastructures.Pair< T1 > |  |
| getValue1() | broad.core.datastructures.Pair< T1 > |  |
| getValue2() | broad.core.datastructures.Pair< T1 > |  |
| hashCode() | broad.core.datastructures.Pair< T1 > |  |
| hasValue1() | broad.core.datastructures.Pair< T1 > |  |
| hasValue2() | broad.core.datastructures.Pair< T1 > |  |
| isEmpty() | broad.core.datastructures.Pair< T1 > |  |
| Pair(T1 v1, T1 v2) | broad.core.datastructures.Pair< T1 > |  |
| Pair() | broad.core.datastructures.Pair< T1 > |  |
| setValue1(T1 v1) | broad.core.datastructures.Pair< T1 > |  |
| setValue2(T1 v2) | broad.core.datastructures.Pair< T1 > |  |


---

Generated on Fri Aug 15 2014 15:10:52 for ESAT by  

 1.8.7
